# PCSK9 Inhibitor and Potential Decreased Risk of Neoplasms, Especially in Females: A Meta-Analysis

**DOI:** 10.3390/ph18081095

**Published:** 2025-07-24

**Authors:** Tingyang Wei, Zonglin Li, Chu Lin, Yuteng Yang, Changjie Tie, Xiaoling Cai, Fang Lv, Wenjia Yang, Linong Ji

**Affiliations:** Department of Endocrinology and Metabolism, Peking University People’s Hospital, Beijing 100044, China; pacisr@163.com (T.W.); 1910301320@pku.edu.cn (Z.L.); chulin@bjmu.edu.cn (C.L.); 2210301231@stu.pku.edu.cn (Y.Y.); 2210305125@stu.pku.edu.cn (C.T.); lvfang1a@163.com (F.L.); ywjsunshine@126.com (W.Y.)

**Keywords:** proprotein convertase subtilisin/kexin type 9 inhibitors, PCSK9i, neoplasm, breast neoplasm, meta-analysis

## Abstract

**Background**: Proprotein convertase subtilisin/kexin type 9 inhibitor (PCSK9i) has been reported to exhibit anti-neoplasm effects. However, the specific impacts remain uncertain. This study aims to evaluate the association between PCSK9i and the risk of neoplasm. **Methods**: Randomized controlled trials (RCTs) comparing PCSK9i with other lipid-lowering drugs or placebo in patients, which reported neoplasm events, were included. Data were sourced from PubMed, Embase, Web of Science, the Cochrane Central Register of Controlled Trials, and the Clinicaltrial.gov website from the inception to June 2024. The primary endpoint was the association between PCSK9i and the risk of overall neoplasm events. **Results**: A total of 37 RCTs with 108,430 participants were included. PCSK9i treatment was associated with a lower risk of neoplasm compared to non-users (RR = 0.92, 95% CI, 0.85 to 0.99, I^2^ = 0%). Subgroup analysis revealed a more prominent risk reduction of overall neoplasm in studies with female-dominant populations (male percentage < 50%, RR = 0.47, 95% CI, 0.27 to 0.82, I^2^ = 0%), with a significant subgroup differences (*p* = 0.02). Meta-regression analysis also suggested that the lower percentage of males was associated with a decreased risk of neoplasms (β = 0.018, 95% CI, 0.0063, 0.031, *p* = 0.002). Meanwhile, the decreased risk of neoplasms was independent of LDL-c reduction. **Conclusions**: PCSK9i therapy was associated with reduced risk of overall neoplasm, especially in female-dominant populations. The benefits for lower risk of neoplasm with PCSK9i treatment were independent of LDL-c reduction.

## 1. Introduction

As a novel lipid-lowering agent recommended by guidelines for adults with hyperlipidemia or at high risk of atherosclerotic cardiovascular disease (ASCVD) [1,2], proprotein convertase subtilisin/kexin type 9 inhibitor (PCSK9i) could reduce serum LDL-C levels by increasing the uptake of low-density lipoprotein cholesterol (LDL-c) in hepatocytes by inhibiting the degradation of low-density lipoprotein receptor (LDLR) in the cell’s surface, which is accelerated by PCSK9 protein [3]. Beyond its established cardiovascular benefits, PCSK9i has drawn increasing attention for potential pleiotropic effects with its widespread application in recent years [4], especially the potential of inhibiting the occurrence and development of neoplasms [5]. A Mendelian randomization study indicated that PCSK9i might reduce the incidence of gastric, hepatic, oral cavity, and pharyngeal cancers and cervical carcinoma in situ [6,7]. Another clinical trial also suggested that Alirocumab would lower the risk of malignant neoplasms in elderly patients with hyperlipidemia [8].

The anti-neoplasm effects of PCSK9i may be partially attributed to its lipid-lowering impact, as most neoplasm cells require high levels of cholesterol for growth [9,10,11,12,13]. However, it was demonstrated that many benefits of PCSK9i were independent of the reduction in LDL-c [4]. In fact, PCSK9 was found to be abnormally highly expressed in a broad spectrum of neoplasms, indicating that PCSK9 might be a promising therapeutic target in neoplasm treatment [14]. PCSK9 can promote immune evasion of neoplasms and act as a promoter for several specific neoplasms [15,16,17]. Animal experiments have also shown that inhibiting PCSK9 can suppress liver metastasis of melanoma in mice and enhance the efficacy of immunotherapy [15,17,18]. Meanwhile, it was observed that overexpression of PCSK9 promoted the development of intestinal adenoma in mice models, while PCSK9i treatment antagonized this process and improved the overall prognosis of mice [19]. Subsequently, PCSK9i may serve as a promising therapeutic strategy for reducing the risks of neoplasms in patients.

However, due to inconsistent evidence from pre-clinical studies and the lack of relevant clinical research, whether PCSK9i treatment could modify the risks of neoplasms remains uncertain. We therefore designed and conducted a comprehensive meta-analysis to clarify the association between PCSK9i treatment and the risk of neoplasms. On this basis, we further explored the influence of different factors, including sex and age, on the anti-neoplasm effect of PCSK9is through subgroup analysis.

## 2. Results

### 2.1. Characteristics and Quality Evaluation of Included Studies

**Figure 1:**
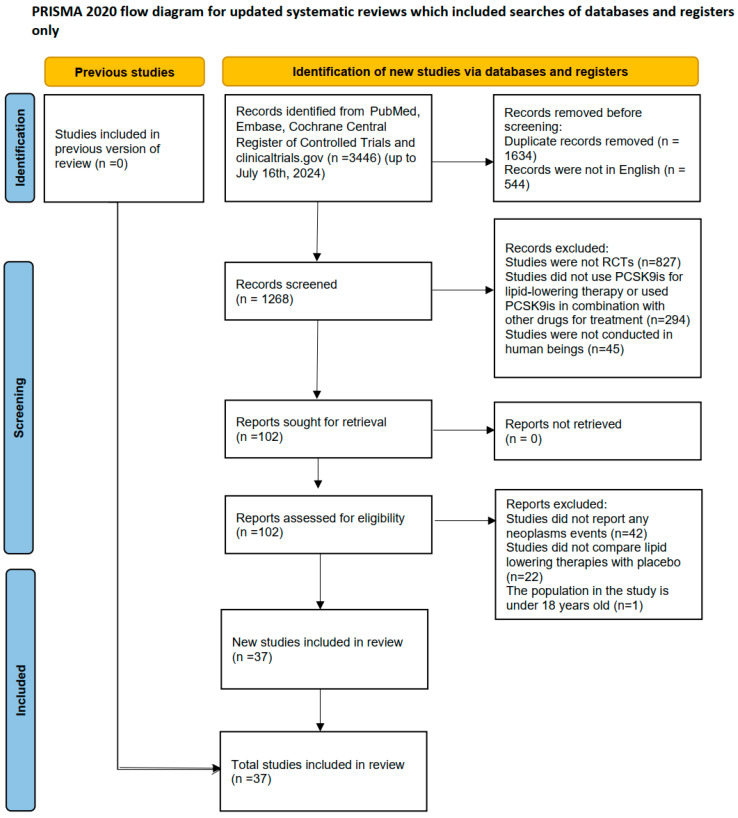
Flowchart of the included studies, including the strategies for literature retrieval and inclusion and exclusion criteria.

A total of 37 RCTs with 108,430 participants were included (Figure 1). The investigated PCSK9is included Alirocumab, Evolocumab, Bococizumab, Olpasiran, and Inclisiran. Baseline characteristics of the included studies are summarized in Table S1. The quality assessments of included studies were performed with Cochrane collaborative tools (Table S2), which suggested a low level of overall risks of bias. Five RCTs had a high risk of bias in allocation concealment, and six RCTs had a high risk of frequent missing outcome data. No RCT was at high risk of selective outcome reporting, randomization sequence generation, bias in masking patients and caregivers, or bias in masking assessors and adjudicators (Table S2). Meanwhile, the funnel plots exhibited even distribution (Figure S1), and the Egger’s test (β = −0.040, 95% CI, −0.131, 0.050, *p* = 0.113) indicated insignificant publication bias, as well.

### 2.2. PCSK9i Treatment and the Risk of Neoplasms

**Figure 2:**
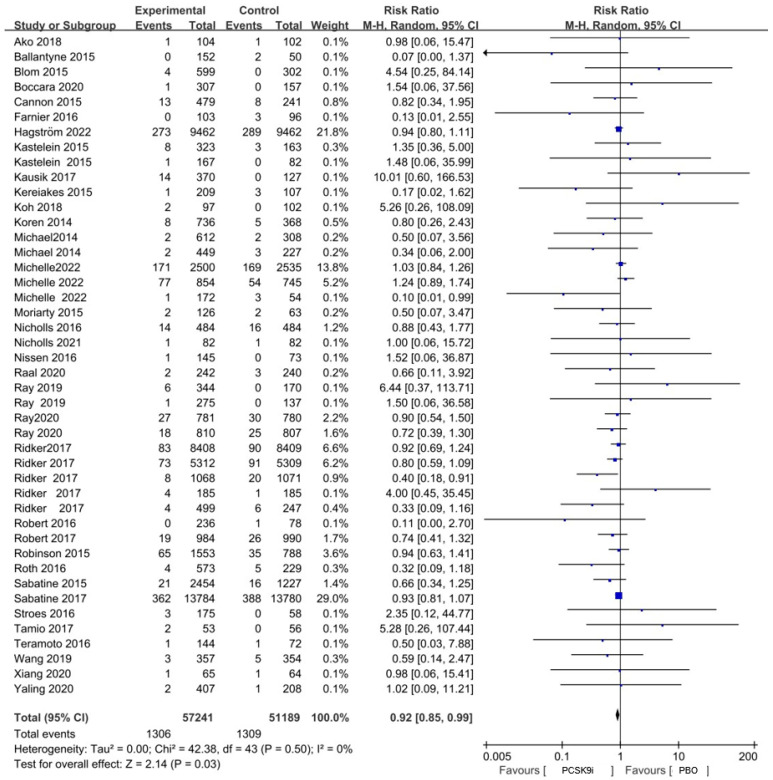
The association between PCSK9i and the incidence of overall neoplasm in the form of a forest plot; the risk ratio is also displayed. Ako 2018 [20]; Ballantyne 2015 [21]; Blom 2015 [22]; Boccara 2020 [23]; Cannon 2015 [24]; Farnier 2016 [25]; Hagström 2022 [26]; Kastelein 2015 [27]; Kausik 2017 [28]; Kereiakes 2015 [29]; Koh 2018 [30]; Koren 2014 [31]; Michael 2014 [31]; Michelle 2022 [32]; Moriarty 2015 [33]; Nicholls 2016 [34]; Nicholls 2021 [35]; Nissen 2016 [36]; Raal 2020 [37]; Ray 2019 [38]; Ray 2020 [39]; Ridker 2017 [40,41]; Robert 2016 [42]; Robert 2017 [43]; Robinson 2015 [44]; Roth 2016 [45]; Sabatine 2015 [46]; Sabatine 2017 [47]; Stroes 2016 [48]; Tamio 2017 [49]; Teramoto 2016 [50]; Wang 2019 [51]; Xiang 2020 [52]; Yaling 2020 [53].

**Figure 3:**
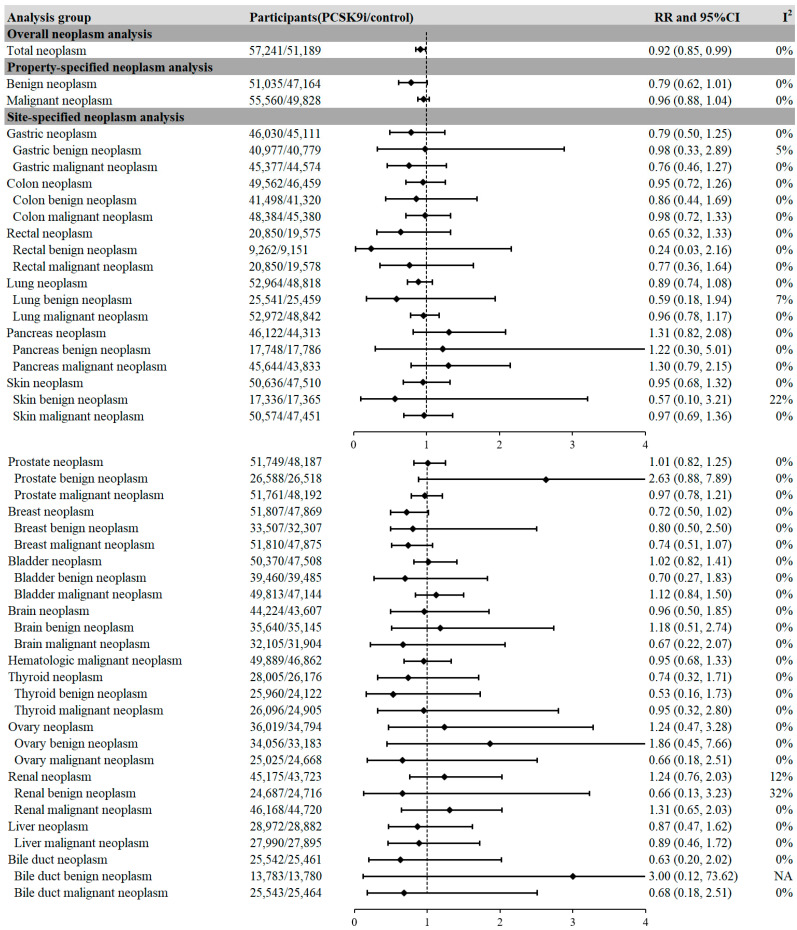

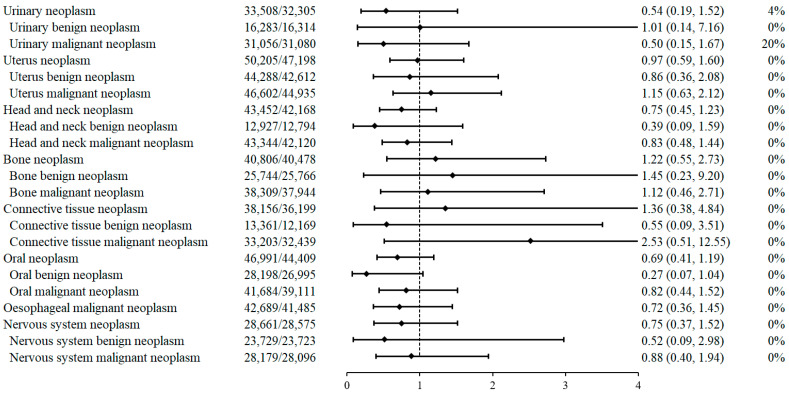
The association between PCSK9i treatment and the incidence of neoplasms in patients. **Legends:** Positive results are presented in bold characters. A reduced risk of total neoplasms was observed in patients receiving PCSK9i.

Overall, compared with non-users, PCSK9i treatment was associated with a lower risk of overall neoplasms in patients (RR = 0.92, 95%CI, 0.85 to 0.99, I^2^ = 0%) (Figure 2 and Figure 3). Based on observed event data, the absolute risk of neoplasm was 2.56% in the control group and 2.28% in the PCSK9i group, resulting in an absolute risk reduction (ARR) of 0.28%. This corresponds to a number needed to treat (NNT) of 357 to prevent one neoplasm case. With respect to the neoplasm sites, when compared with non-PCSK9i users, a modest reduction trend in the risk of breast neoplasm was observed in patients with PCSK9i treatment (RR = 0.72, 95%CI, 0.50 to 1.02, I^2^ = 0%). No significant differences were found between PCSK9i users and non-users in neoplasms concerning other sites and properties (Figure 3).

### 2.3. Factors Associated with Reduced Risk of Neoplasm in PCSK9i Treatment

**Figure 4:**
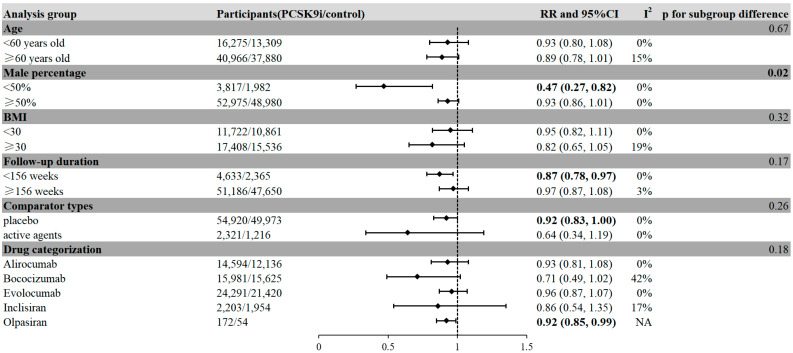
Subgroup analyses of the association between PCSK9i treatment and the incidence of neoplasms in patients. **Legends:** Positive results are presented in bold characters. A reduced risk of neoplasms was observed in female-dominant populations, studies with shorter follow-up durations, and those receiving Olpasiran.

In the subgroup analysis, for grouping of male percentage, a reduced risk of neoplasms was observed in female-dominant populations, defined as studies in which male percentage < 50% (RR = 0.47, 95%CI, 0.27 to 0.82, I^2^ = 0%), with a significant subgroup difference (*p* = 0.02) (Figure 4). None of the other remaining factors, including BMI, follow-up durations, and PCSK9i types, were associated with the risk of neoplasm, or they did not reach significant subgroup differences.

### 2.4. Meta-Regression Analyses

Meta regression analysis indicated that a positive association between male percentage and the risk of neoplasms in patients receiving PCSK9i treatment was found (β = 0.018, 95% CI, 0.0063, 0.031, *p* = 0.002). Furthermore, the risk of neoplasms was not associated with LDL-c reduction in patients with PCSK9i treatment (β = −0.10, 95% CI, −0.34, 0.13, *p* = 0.384). No significant associations were observed with respect to the remaining potentially associated factors (Table S3).

## 3. Discussion

Although the anti-neoplasm potential of PCSK9i has been demonstrated in many pre-clinical studies, as the clinical evidence was not sufficient, the relationship between PCSK9i and the risk of neoplasms in patients remains elusive. According to this meta-analysis of 37 RCTs, we observed a significant association between PCSK9i treatment and a reduced risk of overall neoplasms. Meanwhile, the reduction in neoplasm risk associated with PCSK9i treatment appeared more pronounced in patients with male percentage < 50%. The meta-regression analysis showed a positive association between the percentage of male patients and the risk of neoplasms among those receiving PCSK9i treatment. PCSK9i exhibited promising prospects in lowering the risk of neoplasms, which is worth further elucidation, exploration of the underlying mechanisms, and validation of clinical effects in practice.

In fact, a lot of studies attempting to demonstrate the potential mechanisms for PCSK9i reducing the risk of neoplasms have been carried out. As mentioned earlier, PCSK9is have intensive hypolipidemic effects, and hyperlipidemia was demonstrated to be an important contributor to tumorigenesis [13]. However, according to the meta-regression analysis in our study, no correlations were found between LDL-c reduction and the risk of neoplasms in patients with PCSK9i treatment, which suggested that the anti-neoplasm effects of PCSK9i might be independent of lipid lowering. Previous experiments conducted on melanoma cells with LDL receptor (LDLR) deletion also found that tumor growth inhibition introduced by PCSK9 inhibition was not affected by host LDLR status or cholesterol levels, which is consistent with the notions [16]. Therefore, in addition to reducing serum lipids, PCSK9i may decrease the risk of neoplasms through other specific mechanisms.

The pro-inflammatory effects of PCSK9 in neoplasm environments have been briefly demonstrated before [54]. According to pre-clinical research, after silencing *PCSK9* expression with siRNA, significant reduced *IL-1α*, *IL-6*, and *TNF-α* expression, as well as downregulated activation of the NF-κB pathway, were observed in human THP-1 (an acute monocytic leukemia cell line) cells [54]. By inhibiting PCSK9, the inflammatory process contributing to neoplasm occurrence and progression would be subsequently compromised [55].

In addition to its role in lipid homeostasis, inflammation, and apoptosis, PCSK9 also fundamentally participates in neoplasm immunology, inducing anti-neoplasm effects by accelerating the infiltration of cytotoxic T lymphocytes within the tumor microenvironment and compromising the immune tolerance of neoplasm cells [16]. Correspondingly, PCSK9 inhibition was illustrated to strengthen the treatment effects of cancer immunotherapy. PD-1 blockade is a widely used immunotherapy strategy in neoplasms, and a gene mapping analysis has found the upregulation of PCSK9 in neoplasm cells during the anti-PD-1 process [15]. Pre-clinical studies further elucidated that neutralizing PCSK9 during anti-PD-1 therapy could generate a synergistic anti-tumor effect for colorectal cancer, potentially by promoting CD8+T cell infiltration and inflammatory cytokine release, as well as inhibiting the recruitment of Treg cells [15]. In clinical studies, the application of Evolocumab or Alirocumab was also demonstrated to enhance the anti-tumor effects of anti-PD-1 therapy on several neoplasms, even in patients with cancers with resistance to immune checkpoint therapy [16]. According to our meta-analysis, it was suggested that PCSK9i was associated with a significantly reduced risk of neoplasms. Synthesizing the evidence above, PCSK9i may serve as a promising agent with neoplasm occurrence suppressing effects in patients at high risk of neoplasms through multiple potential mechanisms. Studies to validate this finding and further unravel the underlying mechanisms are required in the future.

Furthermore, in studies with female-predominant populations, the benefits of PCSK9i for reduced risk of neoplasms might be more profound. Many studies have indicated that the risks of neoplasms are profoundly higher in males than females. According to current epidemiological data, the prevalence of lung cancer was 13.3 times higher in males than females [56]. Meanwhile, for liver cancer and pancreatic cancer, the prevalence was 2.9 times and 1.3 times more in male patients than females [57]. Data from the 2017 US Surveillance, Epidemiology, and End Results (SEER) program indicated that cancers of non-reproductive tissues occur more frequently in males than in females, resulting in nearly double the mortality rate of these cancers among men [58]. And, this conclusion has been supported by a large number of studies [59,60,61,62,63]. The sex difference in neoplasm incidence rates is unrelated to race [64]. After accounting for influencing factors, such as diet, exposure to carcinogens, and high-risk behaviors, like smoking and drinking, adult women still demonstrate stronger overall protection against neoplasms compared to men [65,66]. This suggests that the sex differences in cancer incidence may largely stem from distinct cellular and molecular mechanisms inherent to each sex.

The impact of sex differences on the function of PCSK9 has also been supported by existing research [67]. Accumulated evidence has indicated that circulating levels of PCSK9 differ by sex, with females exhibiting significantly higher concentrations than males [68,69,70,71]. These findings suggest that the potential role of PCSK9 may vary by sex, contributing to differences in the anti-cancer effects of PCSK9is.

Such differences may be related to sex hormones and sex chromosomes [59]. Immunity is recognized as a crucial factor in the development of neoplasms, and significant sex differences have been observed, primarily stemming from variations in sex hormones and sex chromosomes [51,72]. From an immune perspective, females exhibit superior immune abilities, characterized by higher levels of immunoglobulin and stronger humoral and cell-mediated immune responses compared to males [73]. This immune mechanism allows female to possess a more robust anti-neoplasm capacity in the early stages of neoplasms [73]. Their tumor microenvironment shows richer infiltration of immune cells, with significant enrichment of dendritic cells, CD4+ T cells, and B cells [73]. This stronger immune response may be a crucial factor contributing to the lower cancer incidence rate in women compared to men [72]. However, this also limits the effectiveness of immune checkpoint inhibitors (ICIs) in females. ICIs work by blocking the immunosuppressive signals used by neoplasm cells, thereby stimulating the body’s immune response [72]. But, this strategy of merely enhancing the immune response has been proven to be not as effective in women who already have higher baseline immune levels compared to men [72]. Studies show that survival HR after immunotherapies in patients with neoplasms are significantly lower in males than females (0.72 versus 0.86, *p* = 0.0019), indicating better therapeutic response in men [74]. However, when they received anti-PD1/anti-PD-L1 plus chemotherapy, women statistically benefited more than men, suggesting that the utilization of anti-neoplasm drugs can enhance the efficacy of immunotherapy in women, potentially becoming a future application scenario for PCSK9is. Nevertheless, these findings are exploratory in nature and may be influenced by ecological confounding inherent to trial-level meta-analyses. The sex difference signal should therefore be viewed as hypothesis-generating, and it calls for individual-level analyses in future studies.

Moreover, because the breast neoplasm occurs principally in female patients, the reduction trend of breast neoplasms in patients receiving PCSK9i may have an impact on the overall effects of PCSK9i on neoplasms in female-predominant populations. More studies concerning sex differences in the anti-neoplasm effects of PCSK9i are required.

According to our research findings, we believe that PCSK9i as a lipid-lowering drug with anti-tumor potential that might serve as a prospective agent for patients requiring lipidemic lowering treatment and at high risk of neoplasms. Meanwhile, the study’s results may also provide inspiration and directions for medicinal development and target selection. Based on the neoplasm risk reducing effects and specific mechanisms of PCSK9 that influence neoplasms’ occurrence and progression from the study’s results and preceding evidence, further agents conforming to similar mechanisms with more specific and concentrated anti-tumor effects are expected to be developed.

However, there are also several limitations to this study. Firstly, due to the variation in study designs, populations, and follow-up durations of the included RCTs, the introduced heterogeneity should not be ignored. To cope with this situation, we conducted further subgroup analyses and meta-regression analyses regarding potential associated baseline characteristics. Secondly, because most included studies were not neoplasm-event-oriented or did not take neoplasms as the study outcome, the reporting of neoplasm events may not be adequately adjudicated, and the study results should therefore be interpreted with caution. Meanwhile, the results of the subgroup analysis were not processed with multiple test adjustments, and the relevant results therefore served as exploratory outcomes, which should be interpreted with caution. Moreover, as the generation and progression of neoplasms are long-term courses, some included studies had relatively short follow-up durations and may not have been able to comprehensively evaluate the occurrence of neoplasms. More studies investigating the anti-tumor effects of PCSK9i over long periods are still required. Because the investigated PCSK9i in enrolled eligible RCTs only comprised antibodies, the effects of PCSK9i in a small interfering ribonucleic acid (siRNA) form on the risk of neoplasms still remain unexplored. Future RCTs investigating PCSK9i in siRNA forms and concentrated on the risks of neoplasms are expected. Finally, this study provides evidence of the association between PCSK9i and the risk of neoplasms in patients, but it did not fully clarify the potential mechanisms. The underlying mechanisms should be further studied. In fact, the anti-tumor effects of statins have been studied and confirmed using in vitro, in vivo, and cell models, which can provide good guidance for the subsequent anti-tumor effects of PCSK9is.

## 4. Materials and Methods

### 4.1. Study Design and Registration

This systematic review and meta-analysis was conducted according to the standards of the Priority Reporting Project for System Evaluation and Meta Analysis (PRISMA) protocol [75]. Registration was accomplished at the International System Evaluation Prospective Registry (PROSPERO) platform with registration number CRD42022376010.

### 4.2. Data Sources and Searches

Conforming to the recommendations of the Cochrane System Evaluation Manual, we conducted a systematic literature search using PubMed, Embase, Web of Science, the Cochrane Central Register of Controlled Trials, and the North American Clinical Trial Registry website, focusing on randomized clinical trials (RCTs) utilizing PCSK9i treatment compared with other lipidemic lowering agents or placebo in patients, which were published between the date of inception (May 2002) and June 2024. Literature searches were implemented through the strategy of using both medical subject headings (MeSH) and free terms. The key words used in the searches were Alirocumab, Bococizumab, Evolocumab, Inclisiran, Olpasiran, PCSK9 inhibitor, hyperlipidemia, dyslipidemia, atherosclerosis, randomized controlled trials, efficacy, and safety. We also screened the relevant research references to further determine all available and eligible RCTs.

### 4.3. Study Selection and Data Extraction

The inclusion criteria of this meta-analysis were (1) RCTs, (2) studies comparing PCSK9i with other non-PCSK9i lipidemic lowering agents or placebo, (3) studies with reports of neoplasm events, and (4) studies conducted with adult patients (≥18 years). Studies were excluded if they (1) had any missing data, (2) were duplicate publications or sub-studies of already included trials, or (3) focused solely on surrogate endpoints (e.g., LDL-C levels) without clinical outcomes.

Two investigators (TW and ZL) independently screened studies by their titles, abstracts, and full texts, excluded the duplicate and ineligible items, evaluated the quality and risk of bias of the remaining studies with the Cochrane risk of bias tool, and abstracted data from eligible studies. The assessment of research quality and bias risk included the following aspects: adequate randomization sequence generation; adequate allocation concealment; blinding of participants and caregivers; binding of outcome assessors and adjudicators; free of infrequent missing outcome data; free of selective outcome reporting; and free of other bias (Table S2). The extracted data included study design, drug exposure, study duration, sample size in experimental groups with PCSK9i treatment, control groups with other active agents or placebo treatment, publication data (first author, published year), baseline characteristics of patients (mean age, body mass index [BMI], sex ratio, race), total neoplasms events, property-specific neoplasm events (benign or malignant), site-specific neoplasm events (the neoplasm sites of interest in this meta-analysis included gastric, colon, rectal, pancreas, hepatic, bile duct, lung, bladder, renal, urinary, prostate, ovary, uterus, breast, cerebrum, hematologic, thyroid, head and neck, bone, skin). Neoplasm events and other data were primarily extracted from the original text or the Supplementary Files attached. The Clinicaltrials.gov website was the subsequent source of neoplasm event data if data were not available in articles and Supplementary Materials. Disagreements were resolved by reaching a consensus with two other joint investigators (CL and XC).

### 4.4. Data Synthesis and Analysis

The primary endpoint of this meta-analysis was the association between PSCK-9i treatment and overall neoplasm event risk in patients, while the secondary endpoint was the risk of benign neoplasms only or malignant neoplasms only among PCSK9i users compared with patients receiving non-PCSK9i treatment. The risk of site-specific neoplasms and subgroup effects based on baseline variables were interpreted as the exploratory endpoint. Results of the meta-analysis were computed as the risk ratio (RR) and 95% confidential intervals (CIs). A random effect model was consistently used due to potential heterogeneity. The heterogeneity included in the study was evaluated using Higgins I^2^ statistics. When the I^2^ value reached ≥50%, a high degree of heterogeneity was indicated. Funnel plots and Egger’s test were used to evaluate publication bias. Statistical significance was considered at *p* < 0.05.

A subgroup analysis was also conducted based on baseline characteristics and other potentially associated factors (including age, sex, BMI, PCSK9i subtype, duration of follow-up, types of comparators) to assess the impact of these cofounders on the effects of PCSK9i on the risk of neoplasms. Meta-regression analyses were also performed to evaluate whether these potential influencing factors were associated with the risk of neoplasms in patients undergoing PCSK9i treatment compared with non-users.

Statistical analysis was conducted using the Review Manager statistical software package (version 5.3, Nordic Cochrane Center, Copenhagen, Denmark) and STATA version 16.0 (STATA, College Station, TX, USA).

## 5. Conclusions

According to this meta-analysis, PCSK9i was associated with significantly decreased risks of overall neoplasms in patients compared with non-users, and the risk reduction might possibly be independent of LDL-c improvement. In particular, our findings highlighted that PCSK9i may exhibit greater benefits towards reduced risks of neoplasms in females, suggesting that PCSK9i could be especially advantageous for female patients. PCSK9i may therefore hold promise as an optimal choice for patients requiring lipidemic control while at high risk of neoplasms, especially for female populations. Further investigations are needed to validate these associations and explore the underlying mechanisms, particularly concerning sex-specific responses to PCSK9i.

## Data Availability

All data relevant to the study are included in the article or uploaded as Supplementary Information. No additional data are available.

## Supplementary Materials

The following supporting information can be downloaded at https://www.mdpi.com/article/10.3390/ph18081095/s1, Figure S1: Funnel plots of publication bias for the included studies; Figure S2: The associations between PCSK9i and the incidences of benign or malignant neoplasm; Table S1: Baseline characteristics of studies included in this meta-analysis; Table S2: Risk of bias in studies included; Table S3: Meta-regression analyses for the association between PCSK9i and the incidence of overall neoplasm in patients. References [20,21,22,23,24,25,27,28,29,30,31,32,33,34,35,36,37,38,39,40,41,42,43,44,45,46,47,48,49,50,52,53,76,77,78,79,80] are cited in the Supplementary Materials.

## Abbreviations

PCSK9i	proprotein convertase subtilisin/kexin type 9 inhibitor
RCT	randomized controlled trial
LDL-c	low-density lipoprotein cholesterol
LDLR	low-density lipoprotein receptor
ASCVD	atherosclerotic cardiovascular disease
BMI	body mass index
MHC I	major histocompatibility complex class I
PD-1	programmed cell death protein 1
PD-L1	programmed death-ligand 1
Treg	regulatory T cell
IL-1α	interleukin-1 alpha
IL-6	interleukin-6
TNF-α	tumor necrosis factor alpha
NF-κB	nuclear factor kappa-light-chain-enhancer of activated B cells
THP-1	human acute monocytic leukemia cell line
NK	natural killer (cell)
HR	hazard ratio
SEER	Surveillance, Epidemiology, and End Results (program)
ICIs	immune checkpoint inhibitors
PRISMA	Preferred Reporting Items for Systematic Reviews and Meta-Analyses
CI	confidence interval
RR	risk ratio
