# Supplementary material for: PCSK9 Inhibitor and Potential Decreased Risk of Neoplasms, Especially in Females: A Meta-Analysis
Source: Pharmaceuticals (Basel). 2025 Jul 24;18(8):1095. doi: 10.3390/ph18081095 (PMC12389645; doi:10.3390/ph18081095)
Supplement: Supplementary file 1 [file pharmaceuticals-18-01095-s001.zip › pharmaceuticals-3665362-supplementary.pdf]

SUPPLEMENTARY MATERIALS

Supplementary Tables

Table S1. Baseline characteristics of studies included in this meta-analysis

Table S2. Risk of bias in studies included

Table S3. Meta-regression analyses for the association between PCSK9i and the incidence of overall neoplasm in patients

Supplementary Figures

Figure S1. Funnel plots of publication bias for the included studies

Figure S2. The associations between PCSK9i and the incidences of benign or malignant neoplasm

Table S1. Baseline characteristics of studies included in this meta-analysis

Author, year	Study duration	Treatment group	No. of patients	Age (years)	Male (%)	BMI (kg/m^2)	Diabetes percentage (%)	CVD percentage (%)	LDL-C change (%)	Baseline LDL-C (mmol/l)
**Alirocumab versus active agents or placebo**										
Robinson, 2015[1]	78 weeks	Alirocumab 150mg	1553	60.4±10.4	63.3	30.2±5.7	0.19	4.6	-52.4±0.9	3.178±1.102
		placebo	788	60.6±10.4	60.2	30.5±5.5	0.25	5.1	3.6±1.3	3.157±1.073
Kereiakes, 2015[2]	24 weeks	Alirocumab 75/Up to 150 mg	209	63.0±9.5	62.7	32.62±6.30	NA	3.38	-42.5±2.5	2.595±0.764
		placebo	107	63.0±8.8	72.0	32.03±7.07	NA	4.67	0.5±3.6	2.746±0.915
Cannon, 2015[3]	112 weeks	Alirocumab 75/Up to 150 mg	479	61.7±9.4	75.2	30.0±5.4	NA	13.99	-48.9±1.7	2.812±0.945
		Ezetimibe	241	61.3±9.2	70.5	30.3±5.1	NA	13.28	-17.0±2.4	2.710±0.884
Farnier, 2016[4]	24 weeks	Alirocumab 75 mg/up to 150 mg + Rosuvastatin 20 mg	54	57.9±8.86	51.9	30.2±6.0	33.3	3.7	-20.6±3.6	3.065±0.834
		Rosuvastatin 20 mg	48	61.5±11.15	68.8	32.0±6.2	58.3	2.08	-8.3±2.4	2.743±0.933
		Alirocumab 75 mg/up to 150 mg + Rosuvastatin 10 mg	49	62.2±11.11	63.3	31.8±7.7	38.8	0	-28.9±2.5	2.78±0.684
		Ezetimibe 10 mg + Rosuvastatin 10 mg	48	60.4±10.38	54.2	32.1 ±7.3	47.9	0	-8.7±2.6	2.653±1.085
Roth, 2016[5]	48 weeks	Alirocumab 75mg	37	59.3 ±11.3	37.8	29.7 ±5.0	10.8	NA	-51.8±2.9	3.842±0.953

		Alirocumab 300mg	146	59.2 ±10.8	45.2	30.9 ±5.7	19.2	NA	-58.4±1.4	3.785±0.868
		placebo	73	59.4 ±10.2	54.8	32.3 ±6.7	23.3	NA	0.3±2.1	3.392±0.787
		Alirocumab 75mg + Statin	78	60.7 ±9.1	65.4	30.1 ±4.9	28.2	NA	-45.3±3.1	2.977±0.933
		Alirocumab 300mg + Statin	312	61.6 ±10.0	60.9	31.4 ±6.2	30.8	NA	-55.3±1.5	2.912±0.85
		placebo + Statin	157	61.6 ±9.7	64.3	30.9 ±6.2	31.8	NA	1.1±2.2	2.903±0.965
Stroes, 2016[6]	24 weeks	Alirocumab 75mg	175	62.5 ±9.9	45.1	29.4 ±5.6	16.6	60	-53.5±1.6	4.002±1.154
		placebo	58	63.1±10.7	53.4	28.5 ±4.6	9	63.8	4.7±2.3	4.106±1.226
Moriarty, 2015[7]	24 weeks	Alirocumab 75 mg/ up to 150 mg	126	64.1±9.0	55.6	29.1 ±5.5	NA	3.17	-45.0±2.2	4.951±1.883
		Atorvastatin	63	63.4±9.5	55.6	NA	NA	1.59	NA	5.011±1.837
Leiter, 2017[8]	24 weeks	Alirocumab 75 mg /Up to 150 mg	344	63.9 ±8.9	54.8	32.6 ±4.8	NA	1.45	-51.8±3.7	3.273±1.506
		placebo	170	64.0 ±9.4	53.1	32.7 ±5.5	NA	2.94	-3.9±5.3	2.853±0.807
Ray, 2019[9]	24 weeks	Alirocumab 75 mg /Up to 150 mg	275	62.8±9.3	53.3	32.7 ±5.4	0	1.82	-43.3±3.6	5.06 ±1.19
		placebo	137	64.1±8.8	50.4	33.2 ±4.9	0.73	0.73	-0.3±4.0	5.25 ±1.32
Schwartz, 2014[10]	64 months	Alirocumab 75 mg /Up to 150 mg	9462	58.5±9.3	74.7	NA	0.41	13.7	NA	NA
		placebo	9462	58.6±9.4	74.9	NA	0.38	15.6	NA	NA
Kastelein, 2015[11]	78 weeks	Alirocumab 75 mg/Up to 150 mg	323	52.1±12.9	55.7	29.0 ± 4.6	9.9	43.0	-48.8±1.6	3.749±1.325

		Placebo	163	51.7±12.3	57.7	30.0 ± 5.4	15.3	43.6	9.1±2.2	3.739±1.213
Kastelein, 2015[11]	78 weeks	Alirocumab 75 mg/up to 150 mg	167	53.2 ± 12.9	51.5	28.6 ± 4.6	4.2	34.1	-48.7±1.9	3.485±1.065
		Placebo	82	53.2 ± 12.5	54.9	27.7 ± 4.7	3.7	29.3	2.8±2.8	3.471±1.071
Teramoto, 2016[12]	52 weeks	Alirocumab 75 mg/Up to 150 mg	144	60.3±9.7	58.3	25.6±4.3	72.9	38.9	-62.5±1.3	3.650±0.693
		Placebo	72	61.8±9.0	65.3	25.4±3.2	59.7	51.3	1.6±1.8	3.668±0.691
Koh, 2018[13]	24 weeks	Alirocumab 75 mg /Up to 150 mg	97	61.2±10.4	61.8	26.3 ±4.0	33	99	-57.1±3.0	2.513±0.721
		placebo	102	60.1±9.1	62.8	26.6 ±3.8	37.3	93.1	6.3±2.9	2.572±0.653
Yaling, 2020[14]	24 weeks	Alirocumab 75 mg /Up to 150 mg	407	58.8 ±10.7	77.4	25.6 ±3.7	29.7	63.4	-56.0±1.5	2.862±1.253
		Ezetimibe	208	58.3 ±11.2	70.2	25.2 ±3.0	23.1	53.4	-20.3±2.0	2.875±1.287
Ako, 2018[15]	9 months	Alirocumab 75 mg	104	61.7±10.9	79.8	NA	NA	7.8	-63.2±1.8	NA
		placebo	102	60.0±11.9	81.4	NA	NA	11.8	-15.5±1.8	NA
Tamio, 2017[16]	64 weeks	Alirocumab 150 mg	53	63.6±10.4	62.3	NA	0	0	-70.1±2.2	3.865±0.806
		Placebo	56	64.6±10.0	66.1	NA	0	1.79	-2.8±2.1	3.870±0.844
**Bococizumab versus active agents or placebo**										
Ballantyne, 2015[17]	24 weeks	Bococizumab 50/100/150mg	152	61.9±9.58	50	32 ± 7	NA	5	-46.8	NA

		placebo	50	60.5±9.84	50	32 ± 7	NA	8.16	-3.6	NA
Ridker, 2017[18]	36 months	Bococizumab 150mg	8394	63.3±9.1	56.9	31.7±6.9	0.05	81.2	NA	NA
		Placebo	8390	63.3±9.2	54.9	31.5±6.9	0	80.9	NA	NA
Ridker, 2017[18]	36 months	Bococizumab 150mg	5281	62.2±9.6	55	30.3±4.7	0.08	81.3	-54.77±0.36	NA
		Placebo	5283	62.5±9.5	59	30±4.5	0.09	79.6	2.13±0.36	NA
Ridker, 2017[19]	52 weeks	Bococizumab 150mg	357	61.1±10.2	55.3	NA	0.28	2.5	-55.6±29.17	NA
		Placebo	354	61.5±9.7	56.7	NA	0	1.7	1.0±20.89	NA
Ridker, 2017[19]	58 weeks	Bococizumab 150mg	1068	61.8±9.3	60.5	NA	0.09	4.7	-54.9±26.84	NA
		Placebo	1071	62.2±9.8	55.7	NA	0.09	3.1	1.0±22.55	NA
Ridker, 2017[19]	52 weeks	Bococizumab 150mg	185	56.5±10.5	59.4	NA	NA	3.8	-54.2±29.34	NA
		Placebo	185	55.7±11.2	59.5	NA	NA	2.7	-0.3±18.30	NA
Ridker, 2017[19]	52 weeks	Bococizumab 150mg	499	61.5±9.9	61.9	NA	NA	2.8	-50.8±29.81	NA
		Placebo	247	61.7±10.0	63.3	NA	NA	4.0	-0.8±17.61	NA
**Evolocumab versus active agents or placebo**										
Koren, 2014[20]	52 weeks	Evolocumab 420mg	882	56.9±11.6	48.0	NA	9.8	20.8	NA	3.6 ±1.0
		Placebo	442	57.6±11.5	45.5	NA	10.1	15.5	NA	3.7 ±1.1

Nissen, 2016[21]	24 weeks	Evolocumab 420mg	145	59.0±11.1	53.8	27.8±4.4	11	33.1	-54.50±1.39	NA
		Ezetimibe	73	58.5±9.4	46.6	28.5±5.9	13.7	28.8	-16.70±1.91	NA
Blom, 2015[22]	52 weeks	Evolocumab 420mg	602	55.9±10.9	48.2	29.9±6.1	10.4	48.2	-50.14±1.24	2.699±0.572
		Placebo	303	56.6±10.3	46.5	30.5±5.9	13.9	49.3	6.83±1.75	2.694±0.559
Sabatine, 2015[23]	52 weeks	Evolocumab 420mg	2454	58.6±10.7	52.1	NA	12.8	51.9	-51.59±28.88	3.108
		Placebo	1227	58.8±10.7	54.4	NA	14.6	52.2	7.43±38.93	3.134
Nicholls, 2016[24]	72 weeks	Evolocumab 420mg	484	59.8±9.6	72.1	29.4±5.0	20.2	82.2	NA	2.398±0.065
		Placebo	486	59.8±8.8	72.2	29.5±5.0	21.5	83.7	NA	2.393±0.062
Sabatine, 2017[25]	26 months	Evolocumab 140mg/420mg	13784	62.5±9.1	75.4	NA	36.7	80.1	NA	2.383
		Placebo	13780	62.5±8.9	75.5	NA	36.5	80.1	NA	2.383
Boccara, 2020[26]	24 weeks	Evolocumab 420mg	307	56.5±9.1	85.5	NA	16.6	50.2	-55.23±1.52	3.452±1.044
		Placebo	157	56.2±8.0	76.4	NA	15.3	43.3	1.68±2.03	3.452±1.036
Nicholls, 2021[27]	48 weeks	Evolocumab 420mg	82	61.1±10.0	75.6	28.2 ± 4.7	16.3	56.3	NA	3.636±0.881
		Placebo	82	60.4±9.4	68.3	28.0 ± 3.9	17.3	40.7	NA	3.68±0.837
Xiang, 2020[28]	12 weeks	Evolocumab 420mg	65	60.0±6.8	40	25.5 ±3.4	NA	19	-59.02±1.82	3.36 ±0.70
		Placebo	64	60.7±7.6	53.1	26.6 ±4.0	NA	7	1.64±1.86	3.68 ±0.68

Michelle, 2022[29]	260 weeks	Evolocumab 140mg/420mg	2500	62.4±8.5	75.1	NA	33.4	82.5	-56.63±36.14	2.383
		Placebo	2535	62.3±8.4	74.8	NA	34.9	84.5	-53.69±37.51	2.357
Michelle, 2022[29]	260 weeks	Evolocumab 140mg/420mg	855	62.6±8.9	82.3	NA	33.4	82.5	-64.72±29.50	2.383
		Placebo	745	62.7±9.1	81.5	NA	34.9	84.5	-66.45±29.84	2.357
Michael, 2014[30]	14 weeks	Evolocumab 140mg	153	52.5±13.7	32	NA	0	35	-57.04±1.23	3.678±0.57
		Ezetimibe biweekly	77	53.9±11.3	31.2	NA	0	25	-17.75±1.67	3.704±0.622
		Placebo biweekly	77	54.4±10.3	36.4	NA	0	16	0.10±1.67	3.626±0.544
		Evolocumab 420mg	153	52.9±12.1	34	NA	0	33	-56.12±1.12	3.73±0.596
		Ezetimibe monthly	77	53.0±12.7	32.5	NA	0	30	-18.57±1.56	3.73±0.596
		Placebo monthly	78	52.6±10.7	39.7	NA	1.3	24	-1.34±1.54	3.73±0.622
Robinson, 2014[31]	144 weeks	Evolocumab 140mg/420mg	1117	59.6±9.9	56	NA	15.7	23.8	NA	2.841±1.096
		Ezetimibe 10mg	221	60.8±9.3	50.7	NA	19.9	17.2	NA	2.833±0.966
		Placebo	558	59.9±10.2	52.2	NA	13.3	22	NA	2.789±1.041
Robert, 2017[32]	12 weeks	Evolocumab 140mg/420mg	984	62.9±8.7	72.7	NA	37.2	86.3	NA	2.357
		Placebo	990	62.7±8.7	72.3	NA	34.3	85.8	NA	2.409
Robert, 2017[33]	12 weeks	Evolocumab 70mg/105mg/140mg	236	59.4±9.9	51.7	28.4	18.2	66.9	-39.06±2.39	3.144±0.632

		Placebo	78	60.2±8.8	46.2	30.1	12	74	2.76±2.39	3.165±0.702
**Inclisiran versus active agents or placebo**										
Ray, 2020[34]	18 months	Inclisiran 284mg	781	66.4±8.9	68.5	NA	47.5	91.4	-56.34	104.5±39.6
		Placebo	780	65.7±8.89	70.3	NA	42.4	89.9	1.30	104.8±37.0
Ray, 2020[34]	17 months	Inclisiran 284mg	810	64.8±8.29	71.5	NA	36.5	79	-49.3	107.2±41.8
		Placebo	807	64.8±8.68	72	NA	33.7	81.9	4.2	103.7±36.4
Raal, 2020[35]	16 months	Inclisiran 300mg	242	54.4±12.48	46.3	NA	8.3	42.1	-41.15	3.921±1.305
		Placebo	240	55.0±11.81	47.9	NA	11.7	42.1	8.37	4.007±1.502
Kausik, 2017[36]	24 weeks	Inclisiran 200/300/500mg	186	63.9±10.8	68.8	NA	NA	NA	-35.39	3.051
		Placebo (Single-dose)	65	62.0±11.4	64.6	NA	NA	NA	1.45	3.328±1.329
		Inclisiran 100/200/300mg	184	62.3±10.8	66.8	NA	NA	NA	-42.59	3.595
		Placebo (Double-dose)	62	65.2±9.4	53.2	NA	NA	NA	0.58	3.243±1.147
**Olpasiran versus active agents or placebo**										
Michelle, 2022[37]	24 weeks	Olpasiran 10/75/225mg	172	61.3±9.2	70.9	NA	31.4	66.3	-0.5	1.943
		placebo	54	63.4±8.9	66.7	NA	22	70	0.23	1.678±0.394

Abbreviations: OAD, oral anti-diabetic agents; BMI, body mass index; NA: not available; CVD, cardiovascular disease; LDL-C, low-density

lipoprotein cholesterol.

Table S2. Risk of bias in studies included

Author, year	Adequate randomization sequence	Adequate allocation concealment	Blinding of participants and caregivers	Binding of outcome assessors and adjudicators	Free of infrequent missing outcome data	Free of selective outcome reporting	Free of other bias

	**generation**						
**Alirocumab versus active agents or placebo**							
**Robinson, 2015[1]**	**Probably yes** Randomized, double-blinded	**Probably yes** Randomized, double-blinded	**Definitely yes**	**Definitely yes**	**Definitely no** Data from 437/1553 (28.1%) participants of experimental group and 193/788 (24.5%) participants of control group missed	**Definitely yes**	**Probably yes** Baseline characteristics were generally balanced
**Kereiakes, 2015[2]**	**Probably yes** Randomized, double-blinded	**Probably yes** Randomized, double-blinded	**Definitely yes**	**Definitely yes**	**Definitely no** Data from 51/209 (24.4%) participants of experimental group and 32/107 (29.9%) participants of control group missed	**Definitely yes**	**Probably yes** Baseline characteristics were generally balanced
**Cannon, 2015[3]**	**Definitely yes** Using an interactive voice response system	**Probably yes** Randomized, double-blinded	**Definitely yes**	**Definitely yes**	**Definitely yes** Data from 0/479 (0%) participants of experimental group and 0/241 (0%) participants of control group missed	**Definitely yes**	**Probably yes** Baseline characteristics were generally balanced
**Farnier, 2016[4]**	**Probably yes** Randomized, double-blinded	**Probably yes** Randomized, double-blinded	**Definitely yes**	**Definitely yes**	**Definitely no** Data from 13/54 (24.1%) participants and 11/49 (22.4%) participants of experimental group and 5/48 (10.4%) participants and 14/48 (29.2%) participants of control group missed	**Definitely yes**	**Probably yes** Baseline characteristics were generally balanced
**Roth, 2016[5]**	**Probably yes** Randomized,	**Probably yes** Randomized,	**Definitely yes**	**Definitely yes**	**Probably no** Data from 103/573	**Definitely yes**	**Probably yes** Baseline

	double-blinded	double-blinded			(18.0%) participants of experimental group and 48/229 (21.0%) participants of control group missed		characteristics were generally balanced
**Stroes, 2016[6]**	**Definitely yes** Using an algorithm	**Probably yes** Randomized, double-blinded	**Definitely yes**	**Definitely yes**	**Probably no** Data from 18/175 (10.3%) participants of experimental group and 4/58 (6.9%) participants of control group missed	**Definitely yes**	**Probably yes** Baseline characteristics were generally balanced
**Moriarty, 2015[7]**	**Probably yes** Randomized, double-blinded	**Probably yes** Randomized, double-blinded	**Definitely yes**	**Definitely yes**	**Definitely no** Data from 30/126 (23.8%) participants of experimental group and 21/63 (33.3%) participants of control group missed	**Definitely yes**	**Probably yes** Baseline characteristics were generally balanced
**Leiter, 2017[8]**	**Probably yes** Randomized, double-blinded	**Definitely yes** double - blind treatment period of 24 weeks	**Definitely yes**	**Definitely yes**	**Probably no** Data from 20/344 (5.8%) participants of experimental group and 10/170 (5.8%) participants of control group missed	**Definitely yes**	**Probably yes** Baseline characteristics were generally balanced
**Ray, 2019[9]**	**Probably yes** Randomized, open-label	**Definitely no** open-label	**Definitely yes**	**Definitely yes**	**Probably no** Data from 31/275 (11.3%) participants of experimental group and 8/137 (5.8%) participants of control group missed	**Definitely yes**	**Probably yes** Baseline characteristics were generally balanced
**Schwartz, 2014[10]**	**Probably yes** Randomized, double-blinded	**Probably yes** Randomized, double-blinded	**Definitely yes**	**Definitely yes**	**Probably no** Data from 2084/9462 (22.0%) participants of experimental group and	**Definitely yes**	**Probably yes** Baseline characteristics were generally

					1515/9462 (16.0%) participants of control group missed		balanced
**Kastelein, 2015[11]**	**Probably yes** Randomized, double-blinded	**Probably yes** Randomized, double-blinded	**Definitely yes**	**Definitely yes**	**Definitely no** Data from 77/323 (23.8%) participants of experimental group and 33/163 (20.2%) participants of control group missed	**Definitely yes**	**Probably yes** Baseline characteristics were generally balanced
**Kastelein, 2015[11]**	**Probably yes** Randomized, double-blinded	**Probably yes** Randomized, double-blinded	**Definitely yes**	**Definitely yes**	**Definitely no** Data from 167/167 (100%) participants of experimental group and 82/82 (100%) participants of control group missed	**Definitely yes**	**Probably yes** Baseline characteristics were generally balanced
**Teramoto, 2016[12]**	**Probably yes** Randomized, double-blinded	**Probably yes** Randomized, double-blinded	**Definitely yes**	**Definitely yes**	**Probably no** Data from 12/144(8.3%) participants of experimental group and 5/102 (8.3%) participants of control group missed	**Definitely yes**	**Probably yes** Baseline characteristics were generally balanced
**Koh, 2018[13]**	**Probably yes** Randomized, double-blinded	**Probably yes** Randomized, double-blinded	**Definitely yes**	**Definitely yes**	**Probably no** Data from 10/97(10.3%) participants of experimental group and 20/156 (12.8%) participants of control group missed	**Definitely yes**	**Probably yes** Baseline characteristics were generally balanced
**Yaling, 2020[14]**	**Probably yes** Randomized, double-blinded	**Definitely yes** Double blind treatment continues until 24 weeks	**Definitely yes**	**Definitely yes**	**Probably no** Data from 25/407(6.1%) participants of experimental group and 17/208 (8.2%) participants of control	**Definitely yes**	**Probably yes** Baseline characteristics were generally balanced

					group missed		
**Ako, 2018[15]**	**Probably yes** Randomized, open-label	**Definitely no** open-label	**Definitely yes**	**Definitely yes**	**Probably no** Data from 10/104(9.6%) participants of experimental group and 8/102 (7.8%) participants of control group missed	**Definitely yes**	**Probably yes** Baseline characteristics were generally balanced
**Tamio, 2017[16]**	**Probably yes** Randomized, open-label	**Definitely no** open-label	**Definitely yes**	**Definitely yes**	**Probably yes** Data from 2/53(3.8%) participants of experimental group and 1/56 (1.8%) participants of control group missed	**Definitely cyes**	**Probably yes** Baseline characteristics were generally balanced
**Bococizumab versus active agents or placebo**							
**Ballantyne, 2015[17]**	**Probably yes** Randomized, double-blinded	**Probably yes** Randomized, double-blinded	**Definitely yes**	**Definitely yes**	**Probably no** Data from 21/152(13.8%) participants of experimental group and 3/50 (6%) participants of control group missed	**Definitely yes**	**Probably yes** Baseline characteristics were generally balanced
**Ridker, 2017[18]**	**Probably yes** Randomized, double-blinded	**Definitely yes** sham dose modifications made to maintain the trial blinding	**Definitely yes**	**Definitely yes**	**Probably yes** Data from 217/8394(2.6%) participants of experimental group and 226/8390 (2.7%) participants of control group missed	**Definitely yes**	**Probably yes** Baseline characteristics were generally balanced
**Ridker, 2017[18]**	**Probably yes** Randomized, double-blinded	**Definitely yes** sham dose modifications made to maintain the trial blinding	**Definitely yes**	**Definitely yes**	**Probably yes** Data from 236/5281 (4.7%) participants of experimental group and 252/5283 (4.8%)	**Definitely yes**	**Probably yes** Baseline characteristics were generally balanced

					participants of control group missed		
**Ridker, 2017[19]**	**Probably yes** Randomized, double-blinded	**Definitely yes** sham dose modifications made to maintain the trial blinding	**Definitely yes**	**Definitely yes**	**Probably no** Data from 43/357(12%) participants of experimental group and 40/354 (11.3%) participants of control group missed	**Definitely yes**	**Probably yes** Baseline characteristics were generally balanced
**Ridker, 2017[19]**	**Probably yes** Randomized, double-blinded	**Definitely yes** sham dose modifications made to maintain the trial blinding	**Definitely yes**	**Definitely yes**	**Probably no** Data from 134/1068(12.5%) participants of experimental group and 146/1071 (13.6%) participants of control group missed	**Definitely yes**	**Probably yes** Baseline characteristics were generally balanced
**Ridker, 2017[19]**	**Probably yes** Randomized, double-blinded	**Definitely yes** sham dose modifications made to maintain the trial blinding	**Definitely yes**	**Definitely yes**	**Probably no** Data from 14/185(7.6%) participants of experimental group and 16/185 (8.6%) participants of control group missed	**Definitely yes**	**Probably yes** Baseline characteristics were generally balanced
**Ridker, 2017[19]**	**Probably yes** Randomized, double-blinded	**Definitely yes** sham dose modifications made to maintain the trial blinding	**Definitely yes**	**Definitely yes**	**Probably no** Data from 74/499(14.8%) participants of experimental group and 29/247(11.7%) participants of control group missed	**Definitely yes**	**Probably yes** Baseline characteristics were generally balanced
**Evolocumab versus active agents or placebo**							
**Koren, 2014[20]**	**Probably yes** Randomized, open-	**Definitely no** open-label	**Definitely yes**	**Definitely yes**	**Probably no** Data from 60/882 (6.8%)	**Definitely yes**	**Probably yes** Baseline

	label				participants of experimental group and 44/442 (10%) participants of control group missed		characteristics were generally balanced
**Nissen, 2016[21]**	**Probably yes** Randomized, double-blinded	**Probably yes** Randomized, double-blinded	**Definitely yes**	**Definitely yes**	**Probably no** Data from 7/145(4.8%) participants of experimental group and 3/73(4.1%) participants of control group missed	**Definitely yes**	**Probably yes** Baseline characteristics were generally balanced
**Blom, 2015[22]**	**Probably yes** Randomized, double-blinded	**Probably yes** Randomized, double-blinded	**Definitely yes**	**Definitely yes**	**Probably no** Data from 34/602(5.6%) participants of experimental group and 16/303(5.3%) participants of control group missed	**Definitely yes**	**Probably yes** Baseline characteristics were generally balanced
**Sabatine, 2015[23]**	**Probably yes** Randomized, open-label	**Definitely no** open-label	**Definitely yes**	**Definitely yes**	**Probably yes** Data from 63/2454(2.6%) participants of experimental group and 30/1227(2.4%) participants of control group missed	**Definitely yes**	**Probably yes** Baseline characteristics were generally balanced
**Nicholls, 2016[24]**	**Definitely yes** Using an interactive voice response system	**Probably yes** Randomized, double-blinded	**Definitely yes**	**Definitely yes**	**Probably yes** Data from 16/484(3.3%) participants of experimental group and 20/486(4.1%) participants of control group missed	**Definitely yes**	**Probably yes** Baseline characteristics were generally balanced
**Sabatine, 2017[25]**	**Definitely yes** Randomization was performed in a double-blinded manner with the use of a central	**Definitely yes** Randomization was performed in a double-blinded manner with the use of a central	**Definitely yes**	**Definitely yes**	**Probably yes** Data from 93/13784(0.7%) participants of experimental group and 118/13780 (0.9%)	**Definitely yes**	**Probably yes** Baseline characteristics were generally balanced

	computerized system	computerized system			participants of control group missed		
**Boccara, 2020[26]**	**Probably yes** Randomized, double-blinded	**Probably yes** Randomized, double-blinded	**Definitely yes**	**Definitely yes**	**Probably yes** Data from 7/307(2.3%) participants of experimental group and 2/157(1.3%) participants of control group missed	**Definitely yes**	**Probably yes** Baseline characteristics were generally balanced
**Nicholls, 2021[27]**	**Probably yes** Randomized, double-blinded	**Probably yes** Randomized, double-blinded	**Definitely yes**	**Definitely yes**	**Probably yes** Data from 3/82(3.7%) participants of experimental group and 6/82(7.3%) participants of control group missed	**Definitely yes**	**Probably yes** Baseline characteristics were generally balanced
**Xiang, 2020[28]**	**Probably yes** Randomized, double-blinded	**Probably yes** Randomized, double-blinded	**Definitely yes**	**Definitely yes**	**Probably yes** Data from 1/65(1.5%) participants of experimental group and 0/64(0%) participants of control group missed	**Definitely yes**	**Probably yes** Baseline characteristics were generally balanced
**Michelle, 2022[29]**	**Probably yes** Randomized, double-blinded	**Probably yes** Randomized, double-blinded	**Definitely yes**	**Definitely yes**	**Probably no** Data from 410/2500 (16.4%) participants of experimental group and 451/2535(17.8%) participants of control group missed	**Definitely yes**	**Probably yes** Baseline characteristics were generally balanced
**Michelle, 2022[29]**	**Probably yes** Randomized, double-blinded	**Probably yes** Randomized, double-blinded	**Definitely yes**	**Definitely yes**	**Probably no** Data from 69/855 (8.1%) participants of experimental group and 50/745(6.7%) participants of control group missed	**Definitely yes**	**Probably yes** Baseline characteristics were generally balanced
**Michael, 2014[30]**	**Probably yes** Randomized,	**Probably yes** Randomized,	**Definitely yes**	**Definitely yes**	**Probably yes** Data from 8/306 (2.6%)	**Definitely yes**	**Probably yes** Baseline

	double-blinded	double-blinded			participants of experimental group and 9/309(2.9%) participants of control group missed		characteristics were generally balanced
**Robinson, 2014[31]**	**Probably yes** Randomized, double-blinded	**Probably yes** Randomized, double-blinded	**Definitely yes**	**Definitely yes**	**Probably no** No reports of missing outcome data were documented in this study	**Definitely yes**	**Probably yes** Baseline characteristics were generally balanced
**Robert, 2017[32]**	**Definitely yes** Using an interactive voice response system	**Probably yes** Randomized, double-blinded	**Definitely yes**	**Definitely yes**	**Probably yes** Data from 31/984(3.2%) participants of experimental group and 22/990(2.2%) participants of control group missed	**Definitely yes**	**Probably yes** Baseline characteristics were generally balanced
**Robert, 2017[33]**	**Definitely yes** Using an interactive voice response system	**Probably yes** Randomized, double-blinded	**Definitely yes**	**Definitely yes**	**Probably yes** Data from 1/236(0.4%) participants of experimental group and 0/78(0%) participants of control group missed	**Definitely yes**	**Probably yes** Baseline characteristics were generally balanced
**Inclisiran versus active agents or placebo**							
**Ray, 2020[34]**	**Probably yes** Randomized, double-blinded	**Probably yes** Randomized, double-blinded	**Definitely yes**	**Definitely yes**	**Probably no** Data from 60/781(7.7%) participants of experimental group and 86/780 (11%) participants of control group missed	**Definitely yes**	**Probably yes** Baseline characteristics were generally balanced
**Ray, 2020[34]**	**Probably yes** Randomized, double-blinded	**Probably yes** Randomized, double-blinded	**Definitely yes**	**Definitely yes**	**Probably yes** Data from 38/810(4.7%) participants of experimental group and 37/807(4.6%) participants of control group missed	**Definitely yes**	**Probably yes** Baseline characteristics were generally balanced

**Raal, 2020[35]**	**Probably yes** Randomized, double-blinded	**Probably yes** Randomized, double-blinded	**Definitely yes**	**Definitely yes**	**Probably yes** Data from 7/242(2.9%) participants of experimental group and 9/240(3.8%) participants of control group missed	**Definitely yes**	**Probably yes** Baseline characteristics were generally balanced
**Kausik, 2017[36]**	**Definitely yes** Using an interactive Web response system	**Probably yes** Randomized, double-blinded	**Definitely yes**	**Definitely yes**	**Probably no** Data from 12/186 (6.5%) participants and 13/184 (7.1%) participants of experimental group and 5/65 (7.7%) participants and 2/62(3.2%) participants of control group missed	**Definitely yes**	**Probably yes** Baseline characteristics were generally balanced
**Olpasiran versus active agents or placebo**							
**Michelle, 2022[37]**	**Probably yes** Randomized, double-blinded	**Probably yes** Randomized, double-blinded	**Definitely yes**	**Definitely yes**	**Probably yes** Data from 8/172 (4.7%) participants of experimental group and 1/54 (1.9%) participants of control group missed	**Definitely yes**	**Probably yes** Baseline characteristics were generally balanced

Table S3. Meta-regression analyses for the association between PCSK9i and the incidence of overall neoplasm in patients

**Parameter**	**β**	**95%CI**	**P value**	**Parameter**	**β**	**95%CI**	**P value**
Age (year old)	-0.004	-0.042, 0.034	0.830	Male percentage (%)	-0.018	-0.006, 0.031	0.004
CAD percentage (%)	-0.003	-0.015, 0.009	0.600	BMI (kg/m^2)	-0.079	-0.216, 0.058	0.239
White race percentage (%)	0.009	-0.005, 0.003	0.023	LDL-c reduction (mmol/L)	-0.102	-0.338, 0.134	0.384

*P<0.05, BMI: body mass index, NA: not available

Figure S1. Funnel plots of publication bias for studies included

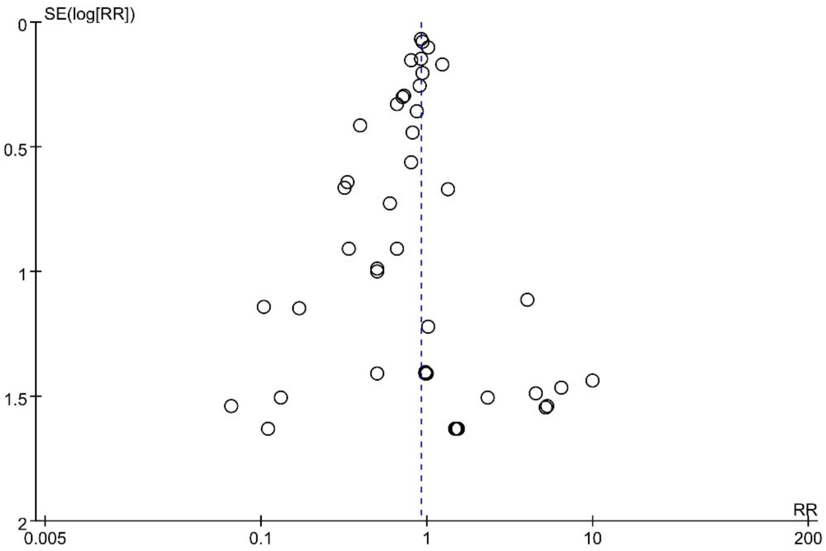

Figure S1-1. Funnel plot of overall studies

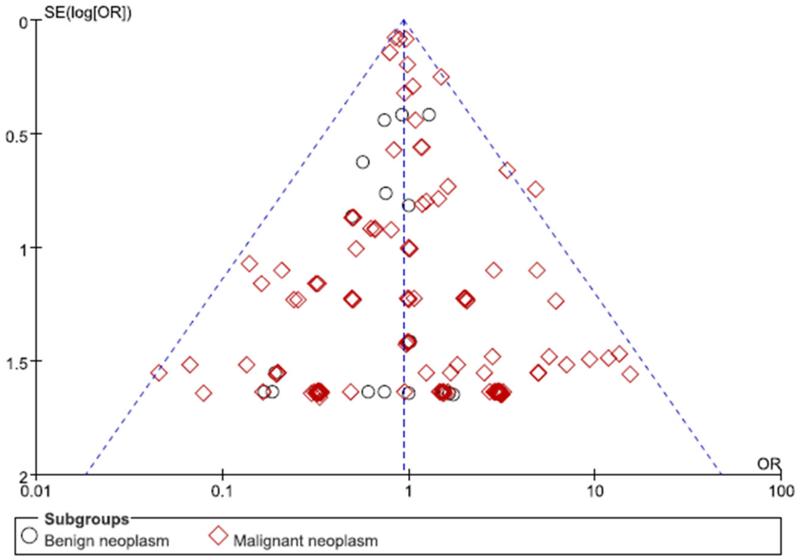

Figure S1-2. Funnel plot of benign/malignant neoplasm subgroup analyses

Figure S2. The associations between PCSK9i and the incidences of benign or malignant neoplasm

	Experimental		Control			Odds Ratio
Study or Subgroup	Events	Total	Events	Total	Weight	M-H, Fixed, 95% CI
**1.3.1 benign**						
Cannon 2015	0	478	0	241		Not estimable
Hagström 2022	21	9441	29	9433	2.3%	0.72 [0.41, 1.27]
Kastelein 2015	1	322	1	162	0.1%	0.50 [0.03, 8.07]
Kausik 2017	2	368	0	127	0.1%	1.74 [0.08, 36.47]
Kereiakes 2015	0	209	1	106	0.2%	0.17 [0.01, 4.16]
Koh 2018	1	96	0	102	0.0%	3.22 [0.13, 80.00]
Koren 2014	1	735	1	367	0.1%	0.50 [0.03, 7.99]
Michael 2014	0	449	1	226	0.2%	0.17 [0.01, 4.12]
Michelle2022	22	2478	22	2513	1.7%	1.01 [0.56, 1.84]
Michelle 2022	8	846	8	737	0.7%	0.87 [0.32, 2.33]
Michelle 2022	1	171	3	51	0.4%	0.09 [0.01, 0.93]
Nicholls 2016	2	482	3	481	0.2%	0.66 [0.11, 3.99]
Ray2020	0	781	1	779	0.1%	0.33 [0.01, 8.16]
Ridker2017	11	8397	15	8394	1.2%	0.73 [0.34, 1.60]
Ridker 2017	3	5309	8	5301	0.6%	0.37 [0.10, 1.41]
Ridker 2017	0	1068	1	1070	0.1%	0.33 [0.01, 8.20]
Robert 2016	4	980	3	987	0.2%	1.34 [0.30, 6.02]
Robert 2017	0	236	1	77	0.2%	0.11 [0.00, 2.67]
Robinson 2015	1	1552	0	788	0.1%	1.52 [0.06, 37.47]
Sabatine 2015	30	13754	30	13750	2.4%	1.00 [0.60, 1.66]
Sabatine 2017	3	2451	4	1223	0.4%	0.37 [0.08, 1.67]
Stroes 2016	2	173	0	58	0.1%	1.71 [0.08, 36.04]
Tamio 2017	2	51	0	56	0.0%	5.71 [0.27, 121.75]
Teramoto 2016	1	143	0	72	0.1%	1.53 [0.06, 37.94]
Xiang 2020	0	65	1	63	0.1%	0.32 [0.01, 7.96]
**Subtotal (95% CI)**		**51035**		**47164**	**11.7%**	**0.79 [0.62, 1.01]**
Total events	116		133			
Heterogeneity: Chi² = 15.22, df = 23 (P = 0.89); I² = 0%						
Test for overall effect: Z = 1.86 (P = 0.06)						
**1.3.2 malignant**						
Ako 2018	1	103	1	101	0.1%	0.98 [0.06, 15.89]
Ballantyne 2015	0	152	2	48	0.3%	0.06 [0.00, 1.29]
Blom 2015	4	595	0	302	0.1%	4.60 [0.25, 85.77]
Boccara 2020	1	306	0	157	0.1%	1.55 [0.06, 38.19]
Cannon 2015	12	467	8	233	0.8%	0.74 [0.30, 1.84]
Farnier 2016	0	103	3	93	0.3%	0.12 [0.01, 2.45]
Hagström 2022	240	9222	257	9205	20.2%	0.93 [0.78, 1.11]
Kastelein 2015	6	317	2	161	0.2%	1.53 [0.31, 7.69]
Kastelein 2015	1	166	0	82	0.1%	1.50 [0.06, 37.11]
Kausik 2017	12	358	0	127	0.1%	9.20 [0.54, 156.51]
Kereiakes 2015	1	208	2	105	0.2%	0.25 [0.02, 2.78]
Koh 2018	1	96	0	102	0.0%	3.22 [0.13, 80.00]
Koren 2014	7	729	4	364	0.4%	0.87 [0.25, 3.00]
Michael2014	2	610	2	306	0.2%	0.50 [0.07, 3.57]
Michael 2014	2	447	1	226	0.1%	1.01 [0.09, 11.21]
Michelle2022	149	2351	147	2388	11.0%	1.03 [0.82, 1.31]
Michelle 2022	69	785	46	699	3.6%	1.37 [0.93, 2.02]
Michelle 2022	1	171	3	51	0.4%	0.09 [0.01, 0.93]
Moriarty 2015	2	124	2	61	0.2%	0.48 [0.07, 3.52]
Nicholls 2016	11	473	13	471	1.0%	0.84 [0.37, 1.89]
Nicholls 2021	1	81	1	81	0.1%	1.00 [0.06, 16.27]
Nissen 2016	1	144	0	73	0.1%	1.54 [0.06, 38.19]
Raal 2020	2	240	3	237	0.2%	0.66 [0.11, 3.96]
Ray 2019	6	338	0	170	0.1%	6.67 [0.37, 119.03]
Ray2020	27	754	28	752	2.2%	0.96 [0.56, 1.65]
Ray 2020	17	793	21	786	1.7%	0.80 [0.42, 1.52]
Ridker2017	69	8339	72	8337	5.7%	0.96 [0.69, 1.33]
Ridker 2017	60	5252	77	5232	6.1%	0.77 [0.55, 1.09]
Ridker 2017	8	1060	19	1052	1.5%	0.41 [0.18, 0.95]
Ridker 2017	3	182	0	185	0.0%	7.23 [0.37, 141.04]
Ridker 2017	4	495	6	241	0.6%	0.32 [0.09, 1.14]
Robert 2016	15	969	22	968	1.7%	0.68 [0.35, 1.31]
Robinson 2015	29	1524	22	766	2.3%	0.66 [0.37, 1.15]
Roth 2016	4	569	2	227	0.2%	0.80 [0.14, 4.38]
Sabatine 2015	328	13456	307	13473	24.1%	1.07 [0.92, 1.25]
Sabatine 2017	14	2440	10	1217	1.1%	0.70 [0.31, 1.57]
Stroes 2016	1	174	0	58	0.1%	1.01 [0.04, 25.17]
Teramoto 2016	0	144	1	71	0.2%	0.16 [0.01, 4.04]
Wang 2019	3	354	5	349	0.4%	0.59 [0.14, 2.48]
Xiang 2020	1	64	0	64	0.0%	3.05 [0.12, 76.21]
Yaling 2020	2	405	1	207	0.1%	1.02 [0.09, 11.34]
**Subtotal (95% CI)**		**55560**		**49828**	**87.8%**	**0.96 [0.88, 1.04]**
Total events	1117		1090			
Heterogeneity: Chi² = 39.60, df = 40 (P = 0.49); I² = 0%						
Test for overall effect: Z = 0.98 (P = 0.33)						
**1.3.3 non-specific**						
Michelle 2022	1	183	0	62	0.1%	1.03 [0.04, 25.55]
Ridker2017	1	8407	3	8406	0.2%	0.33 [0.03, 3.20]
Ridker 2017	2	5310	1	5308	0.1%	2.00 [0.18, 22.06]
Robert 2016	0	984	1	989	0.1%	0.33 [0.01, 8.23]
**Subtotal (95% CI)**		**14884**		**14765**	**0.5%**	**0.68 [0.20, 2.32]**
Total events	4		5			
Heterogeneity: Chi² = 1.41, df = 3 (P = 0.70); I² = 0%						
Test for overall effect: Z = 0.61 (P = 0.54)						
**Total (95% CI)**		**121479**		**111757**	**100.0%**	**0.94 [0.87, 1.02]**
Total events	1237		1228			
Heterogeneity: Chi² = 58.21, df = 68 (P = 0.80); I² = 0%						
Test for overall effect: Z = 1.58 (P = 0.12)						
Test for subgroup differences: Chi² = 2.33, df = 2 (P = 0.31), I² = 14.2%						

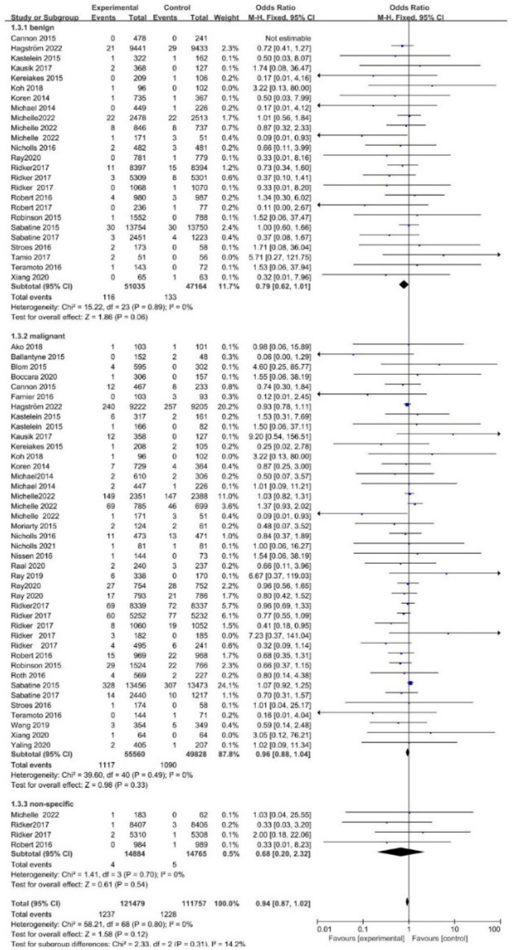

References

1. Robinson, J.G.; Farnier, M.; Krempf, M.; Bergeron, J.; Luc, G.; Averna, M.; Stroes, E.S.; Langslet, G.; Raal, F.J.; El Shahawy, M.; et al. Efficacy and safety of alirocumab in reducing lipids and cardiovascular events. *N Engl J Med* **2015**, *372*, 1489-1499, doi:10.1056/NEJMoa1501031.
2. Kereiakes, D.J.; Robinson, J.G.; Cannon, C.P.; Lorenzato, C.; Pordy, R.; Chaudhari, U.; Colhoun, H.M. Efficacy and safety of the proprotein convertase subtilisin/kexin type 9 inhibitor alirocumab among high cardiovascular risk patients on maximally tolerated statin therapy: The ODYSSEY COMBO I study. *Am Heart J* **2015**, *169*, 906-915.e913, doi:10.1016/j.ahj.2015.03.004.
3. Cannon, C.P.; Cariou, B.; Blom, D.; McKenney, J.M.; Lorenzato, C.; Pordy, R.; Chaudhari, U.; Colhoun, H.M. Efficacy and safety of alirocumab in high cardiovascular risk patients with inadequately controlled hypercholesterolaemia on maximally tolerated doses of statins: the ODYSSEY COMBO II randomized controlled trial. *Eur Heart J* **2015**, *36*, 1186-1194, doi:10.1093/eurheartj/ehv028.
4. Farnier, M.; Jones, P.; Severance, R.; Averna, M.; Steinhagen-Thiessen, E.; Colhoun, H.M.; Du, Y.; Hanotin, C.; Donahue, S. Efficacy and safety of adding alirocumab to rosuvastatin versus adding ezetimibe or doubling the rosuvastatin dose in high cardiovascular-risk patients: The ODYSSEY OPTIONS II randomized trial. *Atherosclerosis* **2016**, *244*, 138-146, doi:10.1016/j.atherosclerosis.2015.11.010.
5. Roth, E.M.; Moriarty, P.M.; Bergeron, J.; Langslet, G.; Manvelian, G.; Zhao, J.; Baccara-Dinet, M.T.; Rader, D.J. A phase III randomized trial evaluating alirocumab 300 mg every 4 weeks as monotherapy or add-on to statin: ODYSSEY CHOICE I. *Atherosclerosis* **2016**, *254*, 254-262, doi:10.1016/j.atherosclerosis.2016.08.043.
6. Stroes, E.; Guyton, J.R.; Lepor, N.; Civeira, F.; Gaudet, D.; Watts, G.F.; Baccara-Dinet, M.T.; Lecorps, G.; Manvelian, G.; Farnier, M. Efficacy and Safety of Alirocumab 150 mg Every 4 Weeks in Patients With Hypercholesterolemia Not on Statin Therapy: The ODYSSEY CHOICE II Study. *J Am Heart Assoc* **2016**, *5*, doi:10.1161/jaha.116.003421.
7. Moriarty, P.M.; Thompson, P.D.; Cannon, C.P.; Guyton, J.R.; Bergeron, J.; Zieve, F.J.; Bruckert, E.; Jacobson, T.A.; Baccara-Dinet, M.T.; Zhao, J.; et al. Efficacy and safety of alirocumab in statin-intolerant patients over 3 years: open-label treatment period of the ODYSSEY ALTERNATIVE trial. *J Clin Lipidol* **2020**, *14*, 88-97.e82, doi:10.1016/j.jacl.2020.01.001.
8. Leiter, L.A.; Cariou, B.; Müller-Wieland, D.; Colhoun, H.M.; Del Prato, S.; Tinahones, F.J.; Ray, K.K.; Bujas-Bobanovic, M.; Domenger, C.; Mandel, J.; et al. Efficacy and safety of alirocumab in insulin-treated individuals with type 1 or type 2 diabetes and high cardiovascular risk: The ODYSSEY DM-INSULIN randomized trial. *Diabetes Obes Metab* **2017**, *19*, 1781-1792, doi:10.1111/dom.13114.
9. Ray, K.K.; Leiter, L.A.; Müller-Wieland, D.; Cariou, B.; Colhoun, H.M.; Henry, R.R.; Tinahones, F.J.; Bujas-Bobanovic, M.; Domenger, C.; Letierce, A.; et al. Alirocumab vs usual lipid-lowering care as add-on to statin therapy in individuals with type 2 diabetes and mixed dyslipidaemia: The ODYSSEY DM-DYSLIPIDEMIA randomized trial. *Diabetes Obes Metab* **2018**, *20*, 1479-1489, doi:10.1111/dom.13257.

10. Schwartz, G.G.; Bessac, L.; Berdan, L.G.; Bhatt, D.L.; Bittner, V.; Diaz, R.; Goodman, S.G.; Hanotin, C.; Harrington, R.A.; Jukema, J.W.; et al. Effect of alirocumab, a monoclonal antibody to PCSK9, on long-term cardiovascular outcomes following acute coronary syndromes: rationale and design of the ODYSSEY outcomes trial. *Am Heart J* **2014**, *168*, 682-689, doi:10.1016/j.ahj.2014.07.028.
11. Kastelein, J.J.; Ginsberg, H.N.; Langslet, G.; Hovingh, G.K.; Ceska, R.; Dufour, R.; Blom, D.; Civeira, F.; Krempf, M.; Lorenzato, C.; et al. ODYSSEY FH I and FH II: 78 week results with alirocumab treatment in 735 patients with heterozygous familial hypercholesterolaemia. *Eur Heart J* **2015**, *36*, 2996-3003, doi:10.1093/eurheartj/ehv370.
12. Teramoto, T.; Kobayashi, M.; Tasaki, H.; Yagyu, H.; Higashikata, T.; Takagi, Y.; Uno, K.; Baccara-Dinet, M.T.; Nohara, A. Efficacy and Safety of Alirocumab in Japanese Patients With Heterozygous Familial Hypercholesterolemia or at High Cardiovascular Risk With Hypercholesterolemia Not Adequately Controlled With Statins - ODYSSEY JAPAN Randomized Controlled Trial. *Circ J* **2016**, *80*, 1980-1987, doi:10.1253/circj.CJ-16-0387.
13. Koh, K.K.; Nam, C.W.; Chao, T.H.; Liu, M.E.; Wu, C.J.; Kim, D.S.; Kim, C.J.; Li, I.; Li, J.; Baccara-Dinet, M.T.; et al. A randomized trial evaluating the efficacy and safety of alirocumab in South Korea and Taiwan (ODYSSEY KT). *J Clin Lipidol* **2018**, *12*, 162-172.e166, doi:10.1016/j.jacl.2017.09.007.
14. Han, Y.; Chen, J.; Chopra, V.K.; Zhang, S.; Su, G.; Ma, C.; Huang, Z.; Ma, Y.; Yao, Z.; Yuan, Z.; et al. ODYSSEY EAST: Alirocumab efficacy and safety vs ezetimibe in high cardiovascular risk patients with hypercholesterolemia and on maximally tolerated statin in China, India, and Thailand. *J Clin Lipidol* **2020**, *14*, 98-108.e108, doi:10.1016/j.jacl.2019.10.015.
15. Ako, J.; Hibi, K.; Kozuma, K.; Miyauchi, K.; Morino, Y.; Shinke, T.; Tsujita, K.; Uno, K.; Kawabata, Y.; Hiro, T. Effect of alirocumab on coronary atheroma volume in Japanese patients with acute coronary syndromes and hypercholesterolemia not adequately controlled with statins: ODYSSEY J-IVUS rationale and design. *J Cardiol* **2018**, *71*, 583-589, doi:10.1016/j.jjcc.2017.11.013.
16. Teramoto, T.; Kondo, A.; Kiyosue, A.; Harada-Shiba, M.; Ishigaki, Y.; Tobita, K.; Kawabata, Y.; Ozaki, A.; Baccara-Dinet, M.T.; Sata, M. Efficacy and safety of alirocumab in patients with hypercholesterolemia not adequately controlled with non-statin lipid-lowering therapy or the lowest strength of statin: ODYSSEY NIPPON study design and rationale. *Lipids Health Dis* **2017**, *16*, 121, doi:10.1186/s12944-017-0513-7.
17. Ballantyne, C.M.; Neutel, J.; Cropp, A.; Duggan, W.; Wang, E.Q.; Plowchalk, D.; Sweeney, K.; Kaila, N.; Vincent, J.; Bays, H. Results of bococizumab, a monoclonal antibody against proprotein convertase subtilisin/kexin type 9, from a randomized, placebo-controlled, dose-ranging study in statin-treated subjects with hypercholesterolemia. *Am J Cardiol* **2015**, *115*, 1212-1221, doi:10.1016/j.amjcard.2015.02.006.
18. Ridker, P.M.; Revkin, J.; Amarenco, P.; Brunell, R.; Curto, M.; Civeira, F.; Flather, M.; Glynn, R.J.; Gregoire, J.; Jukema, J.W.; et al. Cardiovascular Efficacy and Safety of Bococizumab in High-Risk Patients. *N Engl J Med* **2017**, *376*, 1527-1539, doi:10.1056/NEJMoa1701488.
19. Ridker, P.M.; Tardif, J.C.; Amarenco, P.; Duggan, W.; Glynn, R.J.; Jukema, J.W.; Kastelein, J.J.P.; Kim, A.M.; Koenig, W.; Nissen, S.; et al. Lipid-Reduction Variability and

Antidrug-Antibody Formation with Bococizumab. *N Engl J Med* **2017**, *376*, 1517-1526, doi:10.1056/NEJMoa1614062.
20. Koren, M.J.; Giugliano, R.P.; Raal, F.J.; Sullivan, D.; Bolognese, M.; Langslet, G.; Civeira, F.; Somaratne, R.; Nelson, P.; Liu, T.; et al. Efficacy and safety of longer-term administration of evolocumab (AMG 145) in patients with hypercholesterolemia: 52-week results from the Open-Label Study of Long-Term Evaluation Against LDL-C (OSLER) randomized trial. *Circulation* **2014**, *129*, 234-243, doi:10.1161/circulationaha.113.007012.
21. Nissen, S.E.; Stroes, E.; Dent-Acosta, R.E.; Rosenson, R.S.; Lehman, S.J.; Sattar, N.; Preiss, D.; Bruckert, E.; Ceška, R.; Lepor, N.; et al. Efficacy and Tolerability of Evolocumab vs Ezetimibe in Patients With Muscle-Related Statin Intolerance: The GAUSS-3 Randomized Clinical Trial. *Jama* **2016**, *315*, 1580-1590, doi:10.1001/jama.2016.3608.
22. Blom, D.J.; Hala, T.; Bolognese, M.; Lillestol, M.J.; Toth, P.D.; Burgess, L.; Ceska, R.; Roth, E.; Koren, M.J.; Ballantyne, C.M.; et al. A 52-week placebo-controlled trial of evolocumab in hyperlipidemia. *N Engl J Med* **2014**, *370*, 1809-1819, doi:10.1056/NEJMoa1316222.
23. Sabatine, M.S.; Giugliano, R.P.; Wiviott, S.D.; Raal, F.J.; Blom, D.J.; Robinson, J.; Ballantyne, C.M.; Somaratne, R.; Legg, J.; Wasserman, S.M.; et al. Efficacy and safety of evolocumab in reducing lipids and cardiovascular events. *N Engl J Med* **2015**, *372*, 1500-1509, doi:10.1056/NEJMoa1500858.
24. Nicholls, S.J.; Puri, R.; Anderson, T.; Ballantyne, C.M.; Cho, L.; Kastelein, J.J.; Koenig, W.; Somaratne, R.; Kassahun, H.; Yang, J.; et al. Effect of Evolocumab on Progression of Coronary Disease in Statin-Treated Patients: The GLAGOV Randomized Clinical Trial. *Jama* **2016**, *316*, 2373-2384, doi:10.1001/jama.2016.16951.
25. Sabatine, M.S.; Giugliano, R.P.; Keech, A.C.; Honarpour, N.; Wiviott, S.D.; Murphy, S.A.; Kuder, J.F.; Wang, H.; Liu, T.; Wasserman, S.M.; et al. Evolocumab and Clinical Outcomes in Patients with Cardiovascular Disease. *N Engl J Med* **2017**, *376*, 1713-1722, doi:10.1056/NEJMoa1615664.
26. Boccara, F.; Kumar, P.N.; Caramelli, B.; Calmy, A.; López, J.A.G.; Bray, S.; Cyrille, M.; Rosenson, R.S. Evolocumab in HIV-Infected Patients With Dyslipidemia: Primary Results of the Randomized, Double-Blind BEIJERINCK Study. *J Am Coll Cardiol* **2020**, *75*, 2570-2584, doi:10.1016/j.jacc.2020.03.025.
27. Nicholls, S.J.; Kataoka, Y.; Nissen, S.E.; Prati, F.; Windecker, S.; Puri, R.; Hucko, T.; Aradi, D.; Herrman, J.R.; Hermanides, R.S.; et al. Effect of Evolocumab on Coronary Plaque Phenotype and Burden in Statin-Treated Patients Following Myocardial Infarction. *JACC Cardiovasc Imaging* **2022**, *15*, 1308-1321, doi:10.1016/j.jcmg.2022.03.002.
28. Zhang, X.; Stiekema, L.C.A.; Stroes, E.S.G.; Groen, A.K. Metabolic effects of PCSK9 inhibition with Evolocumab in subjects with elevated Lp(a). *Lipids Health Dis* **2020**, *19*, 91, doi:10.1186/s12944-020-01280-0.
29. O'Donoghue, M.L.; Giugliano, R.P.; Wiviott, S.D.; Atar, D.; Keech, A.; Kuder, J.F.; Im, K.; Murphy, S.A.; Flores-Arredondo, J.H.; López, J.A.G.; et al. Long-Term Evolocumab in Patients With Established Atherosclerotic Cardiovascular Disease. *Circulation* **2022**, *146*, 1109-1119, doi:10.1161/circulationaha.122.061620.
30. Koren, M.J.; Lundqvist, P.; Bolognese, M.; Neutel, J.M.; Monsalvo, M.L.; Yang, J.; Kim, J.B.; Scott, R.; Wasserman, S.M.; Bays, H. Anti-PCSK9 monotherapy for hypercholesterolemia: the MENDEL-2 randomized, controlled phase III clinical trial of evolocumab. *J Am Coll Cardiol* **2014**, *63*, 2531-2540,

doi:10.1016/j.jacc.2014.03.018.

31. Robinson, J.G.; Nedergaard, B.S.; Rogers, W.J.; Fialkow, J.; Neutel, J.M.; Ramstad, D.; Somaratne, R.; Legg, J.C.; Nelson, P.; Scott, R.; et al. Effect of evolocumab or ezetimibe added to moderate- or high-intensity statin therapy on LDL-C lowering in patients with hypercholesterolemia: the LAPLACE-2 randomized clinical trial. *Jama* **2014**, *311*, 1870-1882, doi:10.1001/jama.2014.4030.
32. Giugliano, R.P.; Mach, F.; Zavitz, K.; Kurtz, C.; Im, K.; Kanevsky, E.; Schneider, J.; Wang, H.; Keech, A.; Pedersen, T.R.; et al. Cognitive Function in a Randomized Trial of Evolocumab. *N Engl J Med* **2017**, *377*, 633-643, doi:10.1056/NEJMoa1701131.
33. Giugliano, R.P.; Desai, N.R.; Kohli, P.; Rogers, W.J.; Somaratne, R.; Huang, F.; Liu, T.; Mohanavelu, S.; Hoffman, E.B.; McDonald, S.T.; et al. Efficacy, safety, and tolerability of a monoclonal antibody to proprotein convertase subtilisin/kexin type 9 in combination with a statin in patients with hypercholesterolaemia (LAPLACE-TIMI 57): a randomised, placebo-controlled, dose-ranging, phase 2 study. *Lancet* **2012**, *380*, 2007-2017, doi:10.1016/s0140-6736(12)61770-x.
34. Ray, K.K.; Wright, R.S.; Kallend, D.; Koenig, W.; Leiter, L.A.; Raal, F.J.; Bisch, J.A.; Richardson, T.; Jaros, M.; Wijngaard, P.L.J.; et al. Two Phase 3 Trials of Inclisiran in Patients with Elevated LDL Cholesterol. *N Engl J Med* **2020**, *382*, 1507-1519, doi:10.1056/NEJMoa1912387.
35. Raal, F.J.; Kallend, D.; Ray, K.K.; Turner, T.; Koenig, W.; Wright, R.S.; Wijngaard, P.L.J.; Curcio, D.; Jaros, M.J.; Leiter, L.A.; et al. Inclisiran for the Treatment of Heterozygous Familial Hypercholesterolemia. *N Engl J Med* **2020**, *382*, 1520-1530, doi:10.1056/NEJMoa1913805.
36. Ray, K.K.; Landmesser, U.; Leiter, L.A.; Kallend, D.; Dufour, R.; Karakas, M.; Hall, T.; Troquay, R.P.; Turner, T.; Visseren, F.L.; et al. Inclisiran in Patients at High Cardiovascular Risk with Elevated LDL Cholesterol. *N Engl J Med* **2017**, *376*, 1430-1440, doi:10.1056/NEJMoa1615758.
37. O'Donoghue, M.L.; Rosenson, R.S.; Gencer, B.; López, J.A.G.; Lepor, N.E.; Baum, S.J.; Stout, E.; Gaudet, D.; Knusel, B.; Kuder, J.F.; et al. Small Interfering RNA to Reduce Lipoprotein(a) in Cardiovascular Disease. *N Engl J Med* **2022**, *387*, 1855-1864, doi:10.1056/NEJMoa2211023.
